# Ganglionic Long-Term Potentiation in Prehypertensive and Hypertensive Stages of Spontaneously Hypertensive Rats Depends on GABA Modulation

**DOI:** 10.1155/2019/7437894

**Published:** 2019-10-13

**Authors:** Luis A. Martínez, Fredy Cifuentes, Miguel A. Morales

**Affiliations:** Departamento de Biología Celular & Fisiología, Instituto de Investigaciones Biomédicas, Universidad Nacional Autónoma de México, Ciudad de México, Mexico

## Abstract

The sympathetic nervous system (SNS) regulates body functions in normal and pathological conditions and is characterized by the presence of a neuroplastic phenomenon, termed ganglionic long-term potentiation (gLTP). In hypertension, either in spontaneously hypertensive rats (SHR) or in humans, sympathetic hyperfunction, such as elevated SNS outflow and changes in synaptic plasticity have been described. Because enhanced SNS outflow is detected in the hypertensive stage and, more importantly, in the prehypertensive phase of SHR, here we explored whether synaptic plasticity, particularly gLTP, was modified in the superior cervical ganglia (SCG) of prehypertensive SHR. Furthermore, considering that GABA modulates sympathetic synaptic transmission and gLTP in Wistar rats, we studied whether GABA might modulate gLTP expression in SHR. We characterized gLTP in the SCG of young prehypertensive 6-week-old (wo) and adult hypertensive (12 wo) SHR and in the SCG of Wistar Kyoto (WKy) normotensive control rats of the same ages. We found that gLTP was expressed in 6 wo SHR, but not in 12 wo rats. By contrast, in WKy, gLTP was expressed in 12 wo, but not in 6 wo rats. We also found that gLTP depends on GABA modulation, as blockade of GABA-A subtype receptors with its antagonist bicuculline unmasked gLTP expression in adult SHR and young WKy. We propose that (1) activity-dependent changes in synaptic efficacy are altered not only during hypertension but also before its onset and (2) GABA may play a modulatory role in the changes in synaptic plasticity in SHR, because the blockade of GABA-A receptors unmasked the expression of gLTP. These early changes in neuroplasticity and GABA modulation of gLTP could be part of the sympathetic hyperfunction observed in hypertension.

## 1. Introduction

The sympathetic nervous system (SNS) is involved in the regulation of many functions—including blood pressure, cardiac contractility, intestinal motility, and some exocrine gland secretions—under normal [1] and in pathological conditions, such as hypertension [2]. In the SNS, there are some forms of synaptic plasticity that could play a role in controlling the function of innervated organs; thus, they could modify normal functions by enhancing tonic efferent impulses to targets [3]. One of the phenomena of synaptic plasticity present in the SNS is ganglionic long-term potentiation (gLTP), which was described long ago, and is characterized by a long-lasting enhancement in the efficacy of ganglionic transmission induced by a brief high-frequency train of presynaptic impulses [4–6]. The underlying mechanisms are not completely understood. gLTP is a Ca^2+^-dependent phenomenon resulting from the participation of several intracellular signaling pathways [5, 7–12] and may play a role in the modulation of peripheral autonomic nervous system activity, including the regulation of blood vessel tone. The sustained presence of gLTP *in vivo* would reinforce neural outflow to blood vessels, resulting in an increase of peripheral resistance that elevates blood pressure [13].

Hypertension, the chronic elevation of arterial blood pressure, is a major human health problem. Most neurogenic forms of hypertension originate from and are sustained by an increase in sympathetic-adrenal tone [2]. Heightened sympathetic nerve activity has been implicated in the pathophysiology of hypertension observed in animal models and hypertensive patients [14–20]. For example, a loss of spike accommodation and an exaggerated calcium conductance in sympathetic ganglionic neurons from spontaneously hypertensive rats (SHR) compared with that in ganglia of Wistar Kyoto (WKy) normotensive rats has been described [14]. An increase in norepinephrine (NE) turnover in the central nervous system may be the basis of the enhanced sympathetic outflow in hypertensive humans [15, 16]. Magee and Schofield [17] found a larger amplitude of fast excitatory postsynaptic potentials (EPSPs) and currents (EPSCs) in SHR than those in WKy. In addition to the evidence for sympathetic overdrive occurring in hypertension, changes in the neuroplasticity of the SNS have been reported. Magee and Schofield [17] found diminished short-term facilitation in SHR, whereas Alzoubi et al. found that LTP is not expressed in sympathetic ganglia of adult hypertensive SHR [21].

Heightened sympathetic nerve activity can be detected before the development of hypertension both in animal models and in humans [22–25]. For example, an increase in interstitial levels of NE has been found in prehypertensive SHR [23]. Simms et al. [24] reported enhanced respiratory-related bursts of sympathetic activity in neonate and young prehypertensive SHR, while Li et al. [25] found a rise in the depolarization-dependent intracellular free calcium concentration ([Ca^2+^]i) and faster decay of [Ca^2+^]i transients in sympathetic neurons from prehypertensive SHR. In normotensive children of hypertensive parents, sympathetic stimulation triggers a greater increase in plasma levels of NE and endothelin and a greater enhancement of muscle sympathetic nerve activity than it does in normal children whose parents are not hypertensive [22]. Considering these findings, we hypothesized that neuroplastic changes might also occur at earlier ages in SHR. In the present work, we addressed this issue, by characterizing the expression of LTP in the superior cervical ganglia (SCG) of prehypertensive 6-week-old (wo) SHR.

The main classical transmitter of the ganglionic synapse in the SCG is acetylcholine (ACh), which can be colocalized with other classical transmitters, such as GABA [26–30]. The modulatory effect of GABA on ganglionic cholinergic transmission has been described previously [30–33]. GABA acting on GABA-A and GABA-B receptors inhibits ganglionic transmission, and acting on GABA-A receptors also obstructs the occurrence of LTP in the rat SCG [33, 34]. There are other reports on the role of GABA in central and peripheral neural plasticity: Li and Pan [35] showed a neural plasticity of GABA receptor function in neurons of the paraventricular nucleus of SHR. Magnaghi et al. [36] demonstrated that GABA acting on GABA-A and GABA-B receptors controls Schwann-cell proliferation and expression of some specific myelin proteins in sciatic nerve. Likewise, structural changes of synapses in the SCG adult rats after long-term administration of GABA have been reported [37]. We confirmed the contribution of endogenous GABA in the expression of gLTP [30], and reported recently that GABA expression is greater in the SCG of SHR than in WKy [38]. According to the evidence that (i) heightened sympathetic nerve activity can be detected before the development of hypertension in SHR [22–25], (ii) GABA can block the occurrence of LTP in rat SCG of Wistar rats [33, 34], (iii) GABA presence can increase in SCG of SHR [38], and (iv) GABA has an antihypertensive central effect in SHR [39–41], we draw the following hypothesis: “early changes in neuroplasticity, particularly in the expression of gLTP, and its modulation by GABAergic inputs would be part of the mechanisms underlying neurogenic hypertension in SHR” .

In the present study, we explored neuroplastic changes in response to train-evoked gLTP before the onset of and during hypertension. We also characterized the modulatory effects of endogenous GABA in the neuroplastic changes occurring in hypertension.

## 2. Materials and Methods

### 2.1. Animals

Seed stocks of SHR and WKy rats were purchased from Charles River (Boston MA, USA) and then reproduced and bred in the animal house facilities of the Instituto de Fisiología Celular, UNAM. We used 6 wo (90–110 g), 8 wo (180–210 g), and 12 wo (240–270 g) male rats in accordance with the Ethical Guidelines for the Care and Use of Laboratory Animals from the National Academy of Sciences of the United States. The project was approved by the Committee for the Care and Use of Laboratory Animals (CICUAL) of our institute. All efforts were made to minimize the number of animals used, as well as their stress.

### 2.2. Experimental Procedures

Rats were anaesthetized with ketamine (90 mg/kg, i.p.) and xylazine hydrochloride (10 mg/kg, i.p.). The SCG was exposed, rapidly excised, and carefully desheathed. Then, the preganglionic and postganglionic nerve roots were trimmed to a length of 3–5 mm. Next, the ganglia were transferred to a recording chamber (Warner Instruments, Hamden, CT, USA) and perfused with oxygenated (95% O_2_, 5% CO_2_) Krebs-Ringer solution (pH 7.4) containing (in mM) 136 NaCl, 4 KCl, 2 CaCl_2_, 1 MgCl_2_, 1 KH_2_PO_4_, 12 NaHCO_3_, 11 glucose, and 2 *μ*M atropine. All experiments were conducted at a controlled temperature of 24.0 ± 0.5°C to facilitate quantification of gLTP (duration 60–90 min).

For recording and stimulation, we followed a previously described procedure [42]. The preganglionic cervical sympathetic trunk and the internal carotid nerve were pulled into bipolar suction electrodes. Stimuli were applied by a Pulsar 6i Stimulator (FHC, Bowdoin, ME, USA) consisting of supramaximal square voltage test pulses (9–12 V) of 0.1 ms duration at 0.2 Hz. Compound action potentials (CAPs) were recorded from the postganglionic nerve. Voltage traces were amplified (×100), bandpass filtered by a differential amplifier (DP-301; Warner Instruments, Hamden, CT, USA), and digitized with a multifunction data-acquisition board (PCI-DAQ) with a 16-bit A-D converter using a custom-made acquisition program written in LabVIEW (version 8.6; National Instruments, Austin, TX, USA). We measured basal CAP amplitudes over 3 to 4 h to test the stability of the recordings. CAP amplitudes were constant during this period. Differences in baseline transmission between groups were assessed using an input/output (*I*/*O*) curve. To minimize CAP signal saturation, approximately 50%–60% of nicotinic receptors were blocked by the addition of 100 *μ*M of hexamethonium (Sigma-Aldrich, St. Louis, MO, USA) [43]. LTP was induced by a train of supramaximal pulses applied at 40 Hz for 3 s, unless otherwise indicated. The conditioning train produced an immediate increase in the amplitude of postsynaptic CAPs evoked by test pulses. Drugs were applied via Krebs-Ringer solution directly into the recording chamber at the indicated concentrations, between 15 and 40 min before the application of the high-frequency stimulus train, and were maintained during the experiment. All experiments were performed by interleaving the experimental animals with controls to reduce random differences in other factors, distinct from age and strain.

### 2.3. Pharmacological Agents

Stock solution of bicuculline (Sigma-Aldrich, St. Louis, MO, USA) was prepared in dimethyl sulfoxide (DMSO) and stored at -20°C for up to 3 months. Dilutions for experiments were freshly prepared in Krebs-Ringer solution and directly applied into the recording chamber at the indicated final concentrations. The final concentration of DMSO was less than 0.5%, and we verified that this concentration had no effect on synaptic transmission or gLTP.

### 2.4. Data Analysis

To analyze the *I*/*O* curve, we fitted the equation *V*_min_/[1 + ((*V*_max_/*V*_min_) − 1)e^−*aV*^] to data points, where *V*_min_ is the minimum response (ca. 0.003 mV), *V*_max_ is the maximum response, and *a* is the slope coefficient of the curve [44]. The input voltage value that produces half the maximum response (*V*_50_) was estimated by interpolation.

We expressed our data using the relation ΔR/R0, where ΔR = Ri‐R0, Ri is the CAP amplitude at time *t* = *i*, and R0 is the average of the basal CAP amplitude during the 5 min before high-frequency stimulation. The time course of CAP amplitudes recorded in the internal carotid nerve in response to train pulses shows a biexponential decay [4, 5, 10]. Therefore, we fitted the function *f*(*t*) = *αe*^–*t*/*τ*1^ + *ce*^–*t*/*τ*2^ to the data of each experiment. The rapid component (*α* and time constant *τ*1) corresponds to posttetanic potentiation (PTP), while the slow component (*c* and time constant *τ*2) corresponds to gLTP. Using the slow component parameters, we assessed potentiation determining LTP decay as the time in which the ΔR/R0 reaches 20% of potentiation over the amplitude of basal CAPs (ΔR/R0 = 0.2), and LTP extent (area under the curve from *t* = 0 to *t* = LTP decay). For the calculation of LTP extent, we subtracted the area corresponding to the 20% of potentiation [10].

### 2.5. Statistics

Data are expressed as mean ± SEM. The significance level for differences between the means was evaluated with an independent Student *t*-test. The significance level was set at *P* < 0.05.

## 3. Results

**Figure 1:**
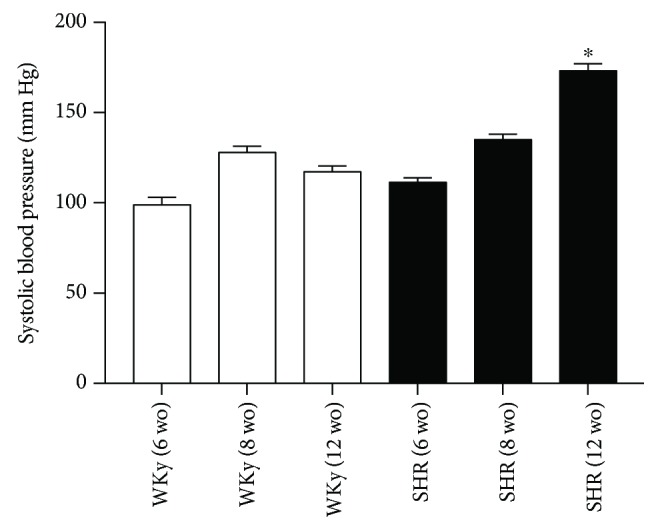
Adult 12 wo SHR showed significant higher blood pressure than 6 and 8 wo SHR and 6, 8, and 12 wo WKy. Systolic blood pressure was (in mm Hg) 111 ± 2 for 6 wo SHR, 135 ± 3 for 8 wo SHR, and 173 ± 4 for 12 wo SHR; for WKy, systolic blood pressure was 98 ± 4 for 6 wo rats, 128 ± 4 for 8 wo rats, and 117 ± 3 for 12 wo rats. Data are mean ± SEM; ^∗^*P* < 0.00001; Student's *t*-test.

The systolic arterial blood pressure was measured before experiments with an indirect tail-cuff apparatus. Six wo SHR and WKy rats were normotensive; 8 wo SHR showed no significant increase in blood pressure, and 12 wo SHR were clearly hypertensive. Systolic arterial blood pressure levels were (in mm Hg) 111 ± 2 for 6 wo SHR, 135 ± 3 for 8 wo SHR, and 173 ± 4 for 12 wo SHR; for WKy, blood pressure levels were 98 ± 4 for 6 wo rats, 128 ± 4 for 8 wo rats, and 117 ± 3 for 12 wo rats; *P* < 0.00001 when comparing 12 wo SHR with the other groups (Figure 1).

### 3.1. Ganglia from Prehypertensive Young SHR Showed Train-Evoked LTP, While Ganglia from Hypertensive Adult SHR Did Not Express Train-Evoked LTP

**Figure 2:**
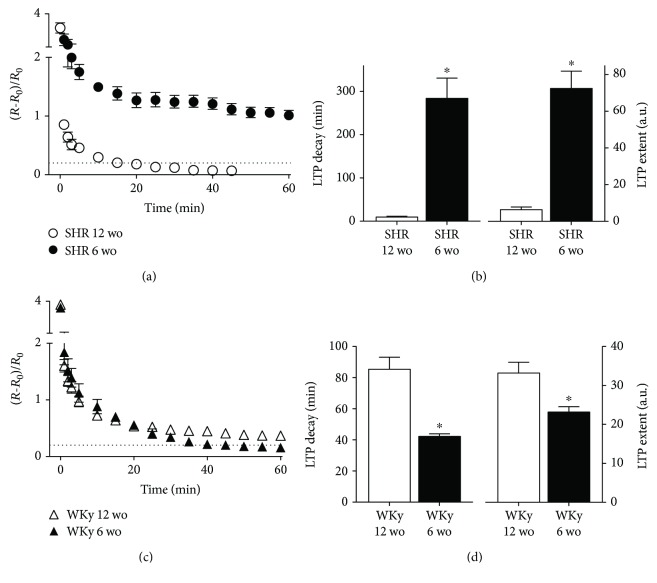
SCG of SHR expressed LTP at 6 wo but not at 12 wo. (a) Time course of synaptic potentiation expressed as ΔR/R0 (mean ± SEM) and recorded in ganglia isolated from young (6 wo) SHR (black circles) and adult (12 wo) hypertensive SHR (white circles). Bar plots of LTP parameters analyzed showing that LTP was expressed in 6 wo SHR, whereas it was not present in 12 wo SHR animals (LTP decay of 284 ± 47 min and LTP extent of 72 ± 22 a.u. for 6 wo SHR (*n* = 5) and LTP decay of 10 ± 2 min and LTP extent of 6 ± 1 a.u. for 12 wo SHR (*n* = 6); *P* = 0.004 and *P* = 0.006, respectively). (c) Time course of synaptic potentiation, ΔR/R0 (mean ± SEM), recorded in ganglia isolated from 6 wo (black triangles) and 12 wo WKy rats (white triangles). (d) LTP was not expressed in SCG of 6 wo WKy rats, but was expressed in 12 wo WKy rats (LTP decay of 42 ± 2 min and LTP extent of 23 ± 1 a.u. for 6 wo WKy rats (*n* = 5) and LTP decay of 85 ± 8 min and LTP extent of 33 ± 3 a.u. for 12 wo WKy rats (*n* = 11); *P* = 0.0002 and *P* = 0.039, respectively). Dotted lines in (a) and (c) indicate the LTP baseline value (0.2).

In SCG from prehypertensive 6 wo SHR, a high-frequency stimulation train of 40 Hz for 3 s evoked a robust LTP (LTP decay of 284 ± 47 min and LTP extent of 72 ± 22 a.u.; Figures 2(a) and 2(b)). By contrast, SCG from adult hypertensive SHR did not show a train-evoked gLTP, the amplitude of the ratio ΔR/R0 decreased rapidly to reach the baseline value (20% potentiation) in less than 15 min after stimulation (LTP decay of 10 ± 2 min and LTP extent of 6 ± 1 a.u.), which was significantly smaller than SCG from 6 wo SHR (*P* = 0.004 and *P* = 0.006 for LTP decay and LTP extent, respectively).

**Figure 3:**
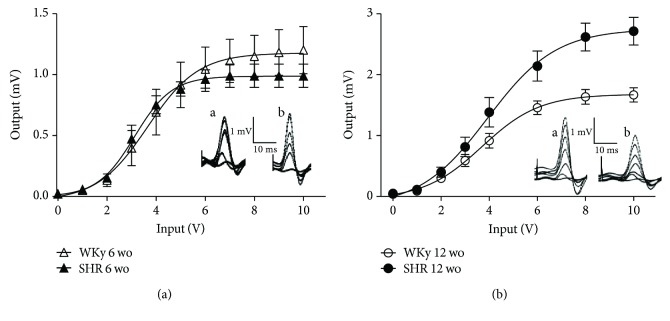
Ganglia from SHR displayed stronger basal transmission at adult age (12 wo) than WKy rats of the same age, while in young (6 wo) rats, transmission was similar in both groups. (a) Input/output curve of ganglionic transmission recorded in the postganglionic nerve of SCG from 6 wo SHR (black triangles: *n* = 5) and age-matched WKy rats (white triangles: *n* = 5). Ganglionic transmission is similar in both groups. (b) Input/output curve of ganglionic transmission recorded in a similar way as in (a) from 12 wo SHR (black circles: *n* = 12) and 12 wo WKy rats (white circles: *n* = 10). Stimuli of similar amplitude evoked a greater response in ganglia from SHR than from WKy rats (*V*_max_ 2.7 ± 0.2 mV vs. 1.7 ± 0.1 mV; SHR vs. WKy; *P* < 0.0007). Insets show sets of compound action potentials (CAPs) from SHR (a) and WKy rats (b) elicited at increasing voltage of stimulation. Note that output scales are different.

To determine the presence of sympathetic hyperactivity, we characterized basal transmission of the SCG from SHR and WKy rats using *I*/*O* curves. We found that 12 wo SHR ganglia exhibited a larger response compared with ganglia from age-matched WKy rats, *V*_max_ 2.7 ± 0.2 mV vs. 1.7 ± 0.1 mV (*P* < 0.0007; Figure 3(b)). We detected that SCG from prehypertensive 6 wo SHR did not show changes in basal transmission compared with WKy rats (Figure 3(a)). Finally, we found that the amplitude of CAPs was smaller in 6 wo than in 12 wo SHR (*V*_max_ 2.7 ± 0.2 for 12 wo vs. 1.0 ± 0.1 for 6 wo; *P* < 0.001).

In WKy rats, we found a different pattern of train-evoked gLTP expression, viz., SCG from 12 wo rats expressed train-evoked LTP (LTP decay of 85 ± 8 min and LTP extent of 33 ± 3 a.u.), whereas ganglia of 6 wo rats expressed a short-lasting train-evoked potentiation; the amplitude of the ratio ΔR/R0 dropped to baseline value in approximately 40 min, with a LTP decay of 42 ± 2 min and a LTP extent of 23 ± 1 a.u., which were significantly less than gLTP from 12 wo WKy rats (*P* = 0.0002 and *P* = 0.039 for LTP decay and LTP extent, respectively; Figures 2(c) and 2(d)).

### 3.2. Ganglia from 8 wo SHR Expressed Evoked LTP

**Figure 4:**
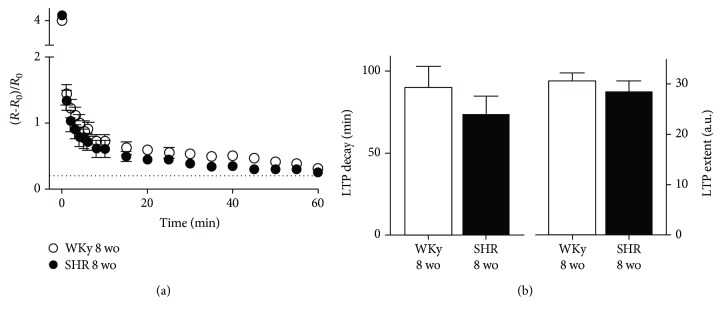
LTP of normal amplitude was expressed in SCG from both SHR and WKy rats at an intermediate age (8 wo). (a) Time course of synaptic potentiation, expressed as ΔR/R0 (mean ± SEM), and recorded in ganglia isolated from SHR (black circles: *n* = 6) and WKy (white circles: *n* = 6) at 8 wo. (b) Bar plots of LTP parameters showing that both SCG from SHR and WKy expressed LTP with values within the ranges of LTP control (LTP decay of 74 ± 11 min and LTP extent of 28 ± 2 a.u. for SHR and LTP decay of 90 ± 13 min and LTP extent of 31 ± 2 a.u. for WKy; *P* > 0.05 for both LTP decay and LTP extent). Dotted line in (a) indicates the LTP baseline (0.2).

Considering the differences observed between young and adult SHR, we explored the expression of evoked LTP in ganglia from SHR with an intermediate age of 8 wo. We found that ganglia from SHR of this age expressed a train-evoked LTP similar to that found in WKy rats (Figure 4). We obtained a LTP decay of 74 ± 11 min and a LTP extent of 28 ± 2 a.u. for SHR and a LTP decay of 90 ± 13 min and a LTP extent of 31 ± 2 a.u. for WKy (*P* > 0.05 for both parameters, LTP decay and LTP extent; Figure 4(b)).

### 3.3. Blockade of GABA-A Subtype Receptors in SCG of Adult SHR Disclosed LTP

**Figure 5:**
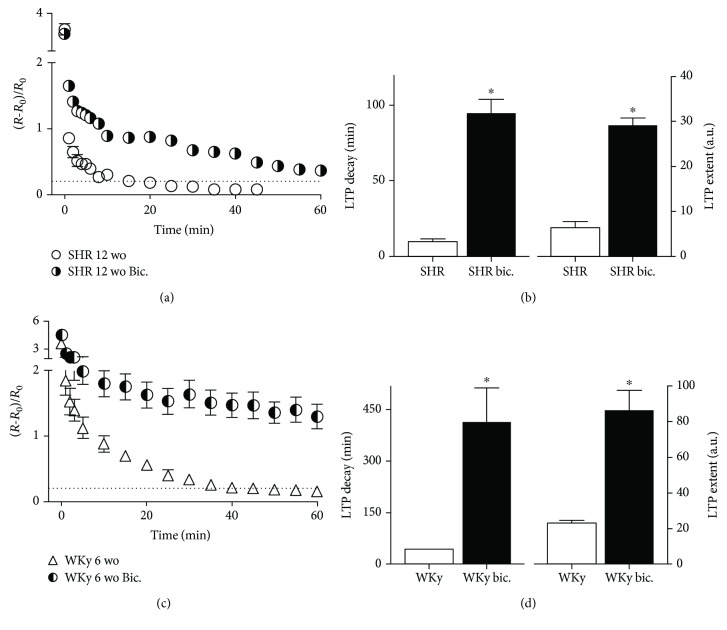
GABA-A receptors modulated LTP in SCG from adult (12 wo) SHR and young (6 wo) WKy rats. (a) Time course of synaptic potentiation, ΔR/R0 (mean ± SEM) recorded in SCG from 12 wo SHR in the absence (white circles) and presence of 2 *μ*M bicuculline (half-full circles). (b) Bar plots of LTP decay and LTP extent showing that LTP was unmasked by GABA-A blockade (LTP decay of 94 ± 9 min and LTP extent of 37 ± 2 a.u. for GABA-A subtype receptor blockade by bicuculline (*n* = 6) and LTP decay of 10 ± 2 min and LTP extent of 6 ± 1 a.u. without bicuculline (*n* = 6); *P* = 0.002 and *P* = 0.004, respectively). (c) Time course of synaptic potentiation, ΔR/R0 (mean ± SEM) recorded in SCG from 6 wo WKy rats in the absence (white triangles) and in the presence of 2 *μ*M bicuculline (half-full circles). (d) Bar plots of LTP decay and LTP extent showing that GABA-A blockade recovered LTP expression of SCG of 6 wo WKy rats (LTP decay of 414 ± 104 min and LTP extent of 86 ± 11 a.u. for GABA blockade by bicuculline (*n* = 5) and LTP decay of 42 ± 2 min and LTP extent of 23 ± 1 a.u. without bicuculline (*n* = 5); *P* = 0.023 and *P* = 0.004, respectively). Dotted lines in (a) and (c) indicate the LTP baseline (0.2).

Taking into account the inhibitory effect of endogenous GABA on gLTP found in Wistar rats [30, 34], we wondered if endogenous GABA has a similar inhibitory effect on gLTP evoked in SHR. To investigate this possibility, we used bicuculline, an antagonist of GABA-A subtype receptor, and found that blockade of likely endogenous GABA inhibition unmasked the appearance of train-evoked LTP in the SCG of adult SHR (Figures 5(a) and 5(b)). In the presence of bicuculline, gLTP was 94 ± 9 min (LTP decay) and LTP extent reached 37 ± 2 a.u., significantly greater than in conditions without bicuculline (*P* = 0.0002 for LTP decay and *P* = 0.004 for LTP extent). Additionally, and considering the short-lasting evoked potentiation found in SCG of 6 wo WKy, we also tested whether endogenous GABA was downregulating LTP at this age. We found that bicuculline indeed unmasked a robust LTP of 414 ± 103 min decay and 86 ± 11 a.u. extent in ganglia from 6 wo WKy rats, values that were significantly larger than the evoked LTP without bicuculline; *P* = 0.023 for LTP decay and *P* = 0.004 for LTP extent (Figures 5(c) and 5(d)).

Considering the frequency range of spontaneous activity of sympathetic preganglionic neurons, 10–20 Hz [45, 46], we characterized evoked gLTP in response to a train of 10 Hz for 12 s. We found that in SHR and WKy rats, this train of stimulation induced gLTP, which reproduced all features found previously using a 40 Hz train. There was no LTP in SCG from 12 wo SHR (LTP decay of 12 ± 3 min and LTP extent of 8 ± 1 a.u.), but there was expression in SCG of 6 wo SHR (LTP decay of 157 ± 17 min and LTP extent of 46 ± 4 a.u.). GABA antagonist unmasked the expression of LTP in SCG from adult SHR (data not shown).

## 4. Discussion

The data presented here demonstrated that gLTP can be evoked in SHR at a young age (6 wo) before the onset of hypertension, and the known lack expression of gLTP in adult (12 wo) SHR can be reverted by antagonizing endogenous ganglionic GABA inhibition.

The expression of evoked gLTP in prehypertensive 6 wo SHR indicates that synaptic plasticity changed before the manifestation of hypertension. Other changes in synaptic plasticity such as reduction in LTP and in short-term facilitation in SCG of adult hypertensive SHR have been described previously [17, 21]. To our knowledge, data reported here are the first evidence that changes in the synaptic plasticity of SCG occurred in SHR at early ages before hypertension develops. Although, there is currently controversy regarding the age of hypertension onset in SHR animals [24, 47], we and others found that 6 wo SHR are normotensive [25, 35, 38, 48].

Our study of ganglionic basal transmission in SHR confirmed that it is increased in adults, as previously reported by Magee and Schofield [17]. By contrast, in juvenile prehypertensive 6 wo SHR, although there was an increase in gLTP, basal ganglionic activity did not increase, as shown in the *I*/*O* curve of ganglia from 6 wo SHR which was not different from the control normotensive 6 wo WKy. However, there are some reports of sympathetic hyperactivity in SHR before the development of hypertension [23–25]. This apparent contradiction may be because ganglionic basal transmission did not increase at 6 wo, but probably later, unlike the other parameters used to assess sympathetic activity.

As expected, adult WKy rats expressed a normal evoked gLTP, while 6 wo WKy were unable to express it. These findings suggest that the mechanisms responsible for gLTP in normal conditions such as in WKy rats require a maturation process that is faster in SHR, where ganglia at 6 wo expressed LTP. In line with this idea, it is known that gLTP is an age-dependent neuroplastic phenomenon [21, 49]. To explore the possibility that gLTP occurs in SHR of an intermediate age, we examined whether ganglia of 8 wo rats were able to express gLTP. We found that at this age gLTP was expressed in SHR, as well as in WKy, which supports the idea of a divergent time course of gLTP expression between SHR and WKy as rats mature.

Our second main finding was the presence of endogenous GABAergic inhibition of gLTP in adult SHR. We found that evoked gLTP in 12 wo SHR was masked by endogenous GABA, because the antagonism of GABA-A receptors by bicuculline unmasked the potentiation phenomenon. In support of this role of GABA, we have recently reported a greater presence of GABA-containing sympathetic varicosities in SCG of 12 wo SHR by comparison with WKy rats [38]. This effect of endogenous GABA might be related with reports of GABAergic antihypertensive effect in SHR. For instance, administration of GABA or the GABA-A receptor agonist muscimol into the lateral brain ventricle or into the hypothalamic paraventricular nucleus lowered mean arterial blood pressure in stroke prone SHR [39–41]. We are aware that bicuculline has actions other than as a GABA-A receptor antagonist [50]; for instance, it also acts on small-conductance calcium-activated potassium channels [51], which are responsible for the afterhyperpolarization in the SCG [52]. However these channels make a small contribution to the nicotinic-evoked ganglionic action potential [53]. More importantly, a lack of this conductance in SCG neurons from SHR has been reported [54]; therefore, it is more likely that bicuculline action is limited to GABA-A receptor blockade.

The findings of an increase in the presence and inhibitory function of GABA in the SCG of SHR were unexpected, taking into account that GABA decreases in the caudal hypothalamus of SHR (sympathetic central nucleus), detected as a reduction in GAD gene expression, resulting in the sympathetic overdrive found in hypertension [55]. A hypothetical explanation for these results is that organisms increase GABA presence in sympathetic ganglia as an effort to counteract the sympathetic overactivity observed in hypertension. An increase in basal transmission was accompanied by a reduction in gLTP; these opposite findings can be explained if we take into account the different cellular mechanisms for LTP and basal transmission (complex and multifactorial process and neurotransmitter release and receptor activation, respectively). It would be expected that an increase in GABA affects mechanisms of gLTP but not those related to basal transmission.

One probable mechanism that might participate in the reduction of LTP in SHR is increasing Cl efflux, similar to the increase found in vascular smooth muscle of the SHR [56]. In line with this assumption, Gonzalez-Burgos et al. [34] found that picrotoxin, a GABA receptor-chloride channel blocker, antagonizes the GABA-mediated inhibition of gLTP.

gLTP was fully expressed in 6 wo SHR, despite the greater presence of GABA [38]. This can be explained by a larger segregation of ACh and GABA detected at 6 wo [38], which would reduce GABA inhibition [30]. By contrast with SHR, in WKy rats, endogenous GABA did not affect the expression of gLTP in 12 wo animals, but did mask the expression at 6 wo. It is possible that in WKy rats, GABA inhibition of gLTP was high at early ages and decreased as rats mature. Similar GABA downregulation during development has been reported in some regions of the central nervous system [57, 58].

Enhancement in gLTP is associated with the development or aggravation of hypertension in various animal models [59, 60]. The increase of gLTP would induce and reinforce sympathetic tone to all target organs, including blood vessels, resulting in an increase in peripheral vessel resistance leading to elevated blood pressure [13]. Alkadhi et al. [59] proposed that the increase in endogenous gLTP in SHR occludes evoked gLTP; however, our data showing unmasking of evoked gLTP by GABA-A subtype receptor blockade by bicuculline challenge this assumption, and suggest that a lack of evoked gLTP in adult SHR is rather due to an increase in endogenous GABA inhibition. Alternative contribution of gLTP to the mechanisms of hypertension has been proposed in other animal models of hypertension, e.g., in the ouabain-dependent model and in the (mRen2)27 transgenic rat model of hypertension, a role for angiotensin II has been suggested [61, 62]. Likewise, in control normotensive rats, 5-HT may play a role in both gLTP and in the control of blood pressure [63]. However, endogenous GABAergic modulation of gLTP was not explored in those studies.

To our knowledge, the present study provides the first evidence that changes in neuroplasticity in the SNS occurred before the onset of the increase in blood pressure, raising the possibility that these changes might be involved in the origin of hypertension. The data also indicate the presence of changes in the modulatory effect of endogenous GABA on gLTP during hypertension and in early ages of normotensive control animals. The early expression of gLTP would imply stronger sympathetic outflow to targets, promoting sustained increase in vascular peripheral resistance.

## Data Availability

Most of the experimental data used to support the findings of this study are included within the article; some others are from previously reported studies, which have been properly cited.

## References

[1] Livingston R. B., West J. B. (1990). Visceral control mechanisms. *Best and Taylor’s Physiological Basis of Medical Practice*.

[2] Guyenet P. G. (2006). The sympathetic control of blood pressure. *Nature Review Neuroscience*.

[3] Cifuentes F., Arias E. R., Morales M. A. (2013). Long-term potentiation in mammalian autonomic ganglia: an inclusive proposal of a calcium-dependent, trans-synaptic process. *Brain Research Bulletin*.

[4] Brown T., McAfee D. (1982). Long-term synaptic potentiation in the superior cervical ganglion. *Science*.

[5] Briggs C. A., Brown T. H., McAfee D. A. (1985). Neurophysiology and pharmacology of long-term potentiation in the rat sympathetic ganglion. *The Journal of Physiology*.

[6] Alonso-deFlorida F., Morales M. A., Minzoni A. A. (1991). Modulated long-term potentiation in the cat superior cervical ganglion in vivo. *Brain Research*.

[7] Alkadhi K. A., Salgado-Commissariat D., Hogan Y. H., Akpaudo S. B. (1996). Induction and maintenance of ganglionic long-term potentiation require activation of 5-hydroxytryptamine (5-HT3) receptors. *The Journal of Physiology*.

[8] Southam E., Charles S. L., Garthwaite J. (1996). The nitric oxide-cyclic GMP pathway and synaptic plasticity in the rat superior cervical ganglion. *British Journal of Pharmacology*.

[9] Hogan Y. H., Hawkins R., Alkadhi K. A. (1998). Adenosine A1 receptor activation inhibits LTP in sympathetic ganglia. *Brain Research*.

[10] Cifuentes F., Licona I. I., De León L., Medina P., De-Miguel F. F., Morales M. A. (2004). Contribution of different calcium channels to long-term potentiation in superior cervical ganglion of the rat. *Neuroscience*.

[11] Vargas R., Cifuentes F., Morales M. A. (2007). Differential contribution of extracellular and intracellular calcium sources to basal transmission and long-term potentiation in the sympathetic ganglion of the rat. *Developmental Neurobiology*.

[12] Vargas R., Cifuentes F., Morales M. A. (2011). Role of presynaptic and postsynaptic IP3-dependent intracellular calcium release in long-term potentiation in sympathetic ganglion of the rat. *Synapse*.

[13] Alkadhi K., Alzoubi K. (2007). Role of long-term potentiation of sympathetic ganglia (gLTP) in hypertension. *Clinical and Experimental Hypertension*.

[14] Yarowsky P., Weinreich D. (1985). Loss of accommodation in sympathetic neurons from spontaneously hypertensive rats. *Hypertension*.

[15] Esler M., Lambert G., Jennings G. (1989). Regional norepinephrine turnover in human hypertension. *Clinical and Experimental Hypertension. Part A: Theory and Practice*.

[16] Esler M., Ferrier C., Lambert G., Eisenhofer G., Cox H., Jennings G. (1991). Biochemical evidence of sympathetic hyperactivity in human hypertension. *Hypertension*.

[17] Magee J. C., Schofield G. G. (1994). Alterations of synaptic transmission in sympathetic ganglia of spontaneously hypertensive rats. *The American Journal of Physiology*.

[18] Segura-Chama P., Hernández A., Jiménez-Pérez N. (2010). Comparison of Ca^2+^ currents of chromaffin cells from normotensive Wistar Kyoto and spontaneously hypertensive rats. *Cellular and Molecular Neurobiology*.

[19] Segura-Chama P., López-Bistrain P., Pérez-Armendáriz E. M., Jiménez-Pérez N., Millán-Aldaco D., Hernández-Cruz A. (2015). Enhanced Ca^2+^-induced Ca^2+^ release from intracellular stores contributes to catecholamine hypersecretion in adrenal chromaffin cells from spontaneously hypertensive rats. *Pflügers Archiv*.

[20] Grassi G. (2016). Sympathomodulatory effects of antihypertensive drug treatment. *American Journal of Hypertension*.

[21] Alzoubi K. H., Aleisa A. M., Alkadhi K. A. (2010). *In vivo* expression of ganglionic long-term potentiation in superior cervical ganglia from hypertensive aged rats. *Neurobiology of Aging*.

[22] Noll G., Wenzel R. R., Schneider M. (1996). Increased activation of sympathetic nervous system and endothelin by mental stress in normotensive offspring of hypertensive parents. *Circulation*.

[23] Cabassi A., Vinci S., Calzolari M., Bruschi G., Borghetti A. (1998). Regional sympathetic activity in pre-hypertensive phase of spontaneously hypertensive rats. *Life Sciences*.

[24] Simms A. E., Paton J. F. R., Pickering A. E., Allen A. M. (2009). Amplified respiratory-sympathetic coupling in the spontaneously hypertensive rat: does it contribute to hypertension?. *The Journal of Physiology*.

[25] Li D., Lee C. W., Buckler K., Parekh A., Herring N., Paterson D. J. (2012). Abnormal intracellular calcium homeostasis in sympathetic neurons from young prehypertensive rats. *Hypertension*.

[26] Wolff J. R., Joó F., Kása P., Storm-Mathiesen J., Toldi J., Balcar V. J. (1986). Presence of neurons with GABA-like immunoreactivity in the superior cervical ganglion of the rat. *Neuroscience Letters*.

[27] Wolff J. R., Kása P., Dobó E., Wenthold R. J., Joo F. (1989). Quantitative analysis of the number and distribution of neurons richly innervated by GABA-immunoreactive axons in the rat superior cervical ganglion. *The Journal of Comparative Neurology*.

[28] Dobó E., Kása P., Wenthold R. J., Joó F., Wolff J. R. (1989). Evidence for GABAergic fibers entering the superior cervical ganglion of rat from the preganglionic nerve trunk. *Histochemistry*.

[29] Ito T., Hioki H., Nakamura K. (2007). Gamma-aminobutyric acid-containing sympathetic preganglionic neurons in rat thoracic spinal cord send their axons to the superior cervical ganglion. *The Journal of Comparative Neurology*.

[30] Elinos D., Rodríguez R., Martínez L. A., Zetina M. E., Cifuentes F., Morales M. A. (2016). Segregation of acetylcholine and GABA in the rat superior cervical ganglia: functional correlation. *Frontiers in Cellular Neuroscience*.

[31] de Groat W. C. (1970). The actions of gamma-aminobutyric acid and related amino acids on mammalian autonomic ganglia. *The Journal of Pharmacology and Experimental Therapeutics*.

[32] Bowery N. G., Brown D. A. (1972). *γ*-Aminobutyrylcholine: actions on GABA and acetylcholine receptors. *The Journal of Pharmacy and Pharmacology*.

[33] González Burgos G. R., Biali F. I., Nicola Siri L. C., Cardinali D. P. (1994). Effect of gamma-aminobutyric acid on synaptic transmission and long-term potentiation in rat superior cervical ganglion. *Brain Research*.

[34] González Burgos G. R., Biali F. I., Cardinali D. P. (1997). Picrotoxin-sensitive receptors mediate gamma-aminobutyric acid-induced modulation of synaptic plasticity in rat superior cervical ganglion. *Brain Research*.

[35] Li D. P., Pan H. L. (2006). Plasticity of GABAergic control of hypothalamic presympathetic neurons in hypertension. *American Journal of Physiology. Heart and Circulatory Physiology*.

[36] Magnaghi V., Ballabio M., Consoli A., Lambert J. J., Roglio I., Melcangi R. C. (2006). GABA receptor-mediated effects in the peripheral nervous system: a cross-interaction with neuroactive steroids. *Journal of Molecular Neuroscience*.

[37] Joó F., Siklós L., Dames W., Wolff J. R. (1987). Fine-structural changes of synapses in the superior cervical ganglion of adult rats after long-term administration of GABA. A morphometric analysis. *Cell and Tissue Research*.

[38] Merino-Jiménez C., Miguel F., Feria Pliego J. A., Zetina Rosales M. E., Cifuentes F., Morales M. A. (2018). Sympathetic hyperactivity and age affect segregation and expression of neurotransmitters. *Frontiers in Cellular Neuroscience*.

[39] Unger T., Becker H., Dietz R. (1984). Antihypertensive effect of the GABA receptor agonist muscimol in spontaneously hypertensive rats. Role of the sympathoadrenal axis. *Circulation Research*.

[40] Allen A. M. (2002). Inhibition of the hypothalamic paraventricular nucleus in spontaneously hypertensive rats dramatically reduces sympathetic vasomotor tone. *Hypertension*.

[41] Akine A., Montanaro M., Allen A. M. (2003). Hypothalamic paraventricular nucleus inhibition decreases renal sympathetic nerve activity in hypertensive and normotensive rats. *Autonomic Neuroscience*.

[42] Arias E. R., Valle-Leija P., Morales M. A., Cifuentes F. (2014). Differential contribution of BDNF and NGF to long-term potentiation in the superior cervical ganglion of the rat. *Neuropharmacology*.

[43] Bachoo M., Polosa C. (1991). Long-term potentiation of nicotinic transmission by a heterosynaptic mechanism in the stellate ganglion of the cat. *Journal of Neurophysiology*.

[44] Banks R. B. (1994). *Growth and Diffusion Phenomena: Mathematical Frameworks and Applications*.

[45] Polosa C. (1968). Spontaneous activity of sympathetic preganglionic neurons. *Canadian Journal of Physiology and Pharmacology*.

[46] Stalbovskiy A. O., Briant L. J. B., Paton J. F. R., Pickering A. E. (2014). Mapping the cellular electrophysiology of rat sympathetic preganglionic neurones to their roles in cardiorespiratory reflex integration: a whole cell recording study in situ. *The Journal of Physiology*.

[47] Dickhout J. G., Lee R. M. (1998). Blood pressure and heart rate development in young spontaneously hypertensive rats. *The American Journal of Physiology*.

[48] Lee R. M., Borkowski K. R., Leenen F. H., Tsoporis J., Coughlin M. (1991). Combined effect of neonatal sympathectomy and adrenal demedullation on blood pressure and vascular changes in spontaneously hypertensive rats. *Circulation Research*.

[49] Wu R., McKenna D. G., McAfee D. A. (1991). Age-related changes in the synaptic plasticity of rat superior cervical ganglia. *Brain Research*.

[50] Johnston G. A. (2013). Advantages of an antagonist: bicuculline and other GABA antagonists. *British Journal of Pharmacology*.

[51] Khawaled R., Bruening-Wright A., Adelman J. P., Maylie J. (1999). Bicuculline block of small-conductance calcium-activated potassium channels. *Pflügers Archiv European Journal of Physiology*.

[52] McAfee D. A., Yarowsky P. J. (1979). Calcium-dependent potentials in the mammalian sympathetic neurone. *The Journal of Physiology*.

[53] Kawai T., Watanabe M. (1986). Blockade of Ca-activated K conductance by apamin in rat sympathetic neurones. *British Journal of Pharmacology*.

[54] Jubelin B. C., Kannan M. S. (1990). Neurons from neonatal hypertensive rats exhibit abnormal membrane properties in vitro. *American Journal of Physiology-Cell Physiology*.

[55] Little H. R., Kramer J. M., Beatty J. A., Waldrop T. G. (2001). Chronic exercise increases GAD gene expression in the caudal hypothalamus of spontaneously hypertensive rats. *Brain Research. Molecular Brain Research*.

[56] Wang Z., Chai Q., Liu Z., Liu D., Chen L. (2004). Chloride channel activity of vascular smooth muscle in the spontaneous hypertensive rats. *Chinese Journal of Physiology*.

[57] Gutiérrez R., Romo-Parra H., Maqueda J. (2003). Plasticity of the GABAergic phenotype of the “glutamatergic” granule cells of the rat dentate gyrus. *The Journal of Neuroscience*.

[58] Somogyi J. (2006). Functional significance of co-localization of GABA and Glu in nerve terminals: a hypothesis. *Current Topics in Medicinal Chemistry*.

[59] Alkadhi K. A., Otoom S. A., Tanner F. L., Sockwell D., Hogan Y. H. (2001). Inhibition of ganglionic long-term potentiation decreases blood pressure in spontaneously hypertensive rats. *Experimental Biology and Medicine*.

[60] Gerges N. Z., Aleisa A. M., Alhaider A. A., Alkadhi K. A. (2002). Reduction of elevated arterial blood pressure in obese Zucker rats by inhibition of ganglionic long-term potentiation. *Neuropharmacology*.

[61] Aileru A. A., De Albuquerque A., Hamlyn J. M. (2001). Synaptic plasticity in sympathetic ganglia from acquired and inherited forms of ouabain-dependent hypertension. *American Journal of Physiology. Regulatory, Integrative and Comparative Physiology*.

[62] Aileru A. A., Logan E., Callahan M., Ferrario C. M., Ganten D., Diz D. I. (2004). Alterations in sympathetic ganglionic transmission in response to angiotensin II in (mRen2)27 transgenic rats. *Hypertension*.

[63] Alkadhi K., Alzoubi K., Aleisa A. (2005). Plasticity of synaptic transmission in autonomic ganglia. *Progress in Neurobiology*.

